# Three-Dimensional Reconstruction of Bacteria with a Complex Endomembrane System

**DOI:** 10.1371/journal.pbio.1001565

**Published:** 2013-05-21

**Authors:** Rachel Santarella-Mellwig, Sabine Pruggnaller, Norbert Roos, Iain W. Mattaj, Damien P. Devos

**Affiliations:** 1 European Molecular Biology Laboratory, Heidelberg, Germany; 2 Department of Molecular Biosciences, University of Oslo, Oslo, Norway; University of California Davis, United States of America

## Abstract

The division of cellular space into functionally distinct membrane-defined compartments has been one of the major transitions in the history of life. Such compartmentalization has been claimed to occur in members of the *Planctomycetes*, *Verrucomicrobiae*, and *Chlamydiae* bacterial superphylum. Here we have investigated the three-dimensional organization of the complex endomembrane system in the planctomycete bacteria *Gemmata obscuriglobus*. We reveal that the *G. obscuriglobus* cells are neither compartmentalized nor nucleated as none of the spaces created by the membrane invaginations are closed; instead, they are all interconnected. Thus, the membrane organization of *G. obscuriglobus*, and most likely all PVC members, is not different from, but an extension of, the “classical” Gram-negative bacterial membrane system. Our results have implications for our definition and understanding of bacterial cell organization, the genesis of complex structure, and the origin of the eukaryotic endomembrane system.

## Abstract 2

The apparently complex membrane organization of *Gemmata obscuriglobus*, and probably all PVC superphylum members, comprises interconnected invaginations and is topologically identical to the “classical” Gram-negative bacterial membrane system.

## Author Summary

The compartmentalization of cellular space has been an important evolutionary innovation, allowing for the functional specialization of cellular space. This compartmentalization is extensively developed in eukaryotes and although not as complex and developed, compartments with specialized function are known to occur in bacteria and can be surprisingly sophisticated. Nevertheless, members of the *Planctomycetes, Verrucomicrobiae*, and *Chlamydiae* (PVC) bacterial superphylum are exceptional in displaying diverse and extensive intracellular membranous organization. We investigated the three-dimensional organization of the complex endomembrane system in the planctomycete bacterium *Gemmata obscuriglobus*. We reveal that the *G. obscuriglobus* cells are neither compartmentalized nor nucleated, contrary to previous claims, as none of the spaces created by the membrane invaginations is topologically closed; instead, they are all interconnected. The organization of cellular space is similar to that of a classical Gram-negative bacterium modified by the presence of large invaginations of the inner membrane inside the cytoplasm. Thus, the membrane organization of *G. obscuriglobus*, and most likely all PVC members, is not fundamentally different from, but is rather an extension of, the “classical” Gram-negative bacterial membrane system.

## Introduction

The compartmentalization of cellular space has been an important evolutionary innovation, allowing for the functional specialization of the membrane-bound organelles. This compartmentalization is extensively developed in eukaryotes, and although not as complex and developed, compartments with specialized function are known to occur in bacteria [1]. Some examples include protein-bound organelles, like carboxysomes, which increase the concentration of metabolite in a closed space [2] and gas vesicles, which are gas-filled protein-bound organelles that function to modulate the buoyancy of cells [3]. Other examples include the magnetosomes in magnetotactic bacteria, which are invaginations of the cytoplasmic membrane that enclose a magnetic mineral without achieving separation into individual vesicles [4]. Individual magnetosomes are arranged into one or more chains within the cell, where they act to orient the cell within a magnetic field. Photosynthetic prokaryotes including the purple bacteria, the cyanobacteria, and the green bacteria have photosynthetic membranes extending from their inner membrane (IM), also called cytoplasmic membrane, maximizing the size of the membrane surface exposed to light. These membranes can adopt diverse shapes, including the formation of membrane stacks continuous with the cell membrane, spherical invaginations of the inner membrane so that multiple membrane spheres are connected to one another or are folded in an accordion-like structure and adjacent to the cell membrane [5]. Lastly, the anammoxosome is a membrane-bound compartment found in the anammox bacteria, which are divergent planctomycetes. It houses the anaerobic ammonium oxidation reaction. Its membrane is enriched in unusual concatenated lipids, the ladderane lipids, which form an impermeable barrier preventing the diffusion of the toxic intermediates produced during the anammox reaction [6].

Bacterial cell organization can be surprisingly complex. Nevertheless, members of the *Planctomycetes, Verrucomicrobiae,* and *Chlamydiae* (PVC) bacterial superphylum are exceptional in displaying diverse and extensive intracellular membranous organization. For this reason they have been labeled the “compartmentalized bacteria” [7],[8]. The planctomycete *Gemmata obscuriglobus* is particularly interesting because a double membrane, formed from a folded single membrane, has been suggested to surround its genetic material. This double membrane is reminiscent of the eukaryotic nuclear envelope, leading to the name “nucleated bacterium” [7],[9]. Early ultrastructural analysis based on thin sections of cryo-substituted cells, freeze-fracture replicas, and computer-aided 3-D reconstructions has been used to argue that the DNA in *G. obscuriglobus* is enclosed within a compartment separated from the rest of the cytoplasm [8],[10]. However, the data are not entirely convincing. A three-dimensional (3D) reconstruction from serial sections and fluorescence microscopy of living cells was presented to support the claim of “the continuous nature of the membranous envelope surrounding the nuclear body and completely enclosing the nucleoid, apart from where gaps appear in the envelope” [8]. As stated by the authors, the “outer region of the nuclear body has a similar appearance to the cytoplasm,” and ribosomes are located in the same compartment as the DNA, arguing against the specific nature of this compartment. In addition, ribosomes line the walls of the internal membrane of the “nuclear compartment” [8], as observed along the inner membrane (IM) of classical bacteria.

This and other analyses have led to the suggestion that the PVC cell plan is different from “classical” Gram-negative bacteria, such as *E. coli*, because of the absence of a typical outer membrane (OM) [7],[8]. The outermost membrane closely juxtaposed to the cell wall was interpreted as the cytoplasmic membrane, while the remaining membrane was called the intracytoplasmic membrane (ICM), mainly based on the distinctive organization of the ICM supposedly surrounding the DNA. The claimed absence of an OM implied the absence of a periplasm, the volume located between IM and OM in Gram-negative bacteria. More recent evidence based on genomic information argue against this conclusion, including the presence of genes associated with the OM and the periplasm in Gram-negative bacteria [11],[12], and the presence of remnants of the division cluster and the peptidoglycan synthesis pathway (typically anchored in the OM) [13].

A more recent analysis of vitrified sections by cryo-electron tomography implied that the “internal membrane” system might be continuous with the ICM, but formed by membrane invaginations and that “the bacterial nucleoid is not completely sealed by the double-membrane system” [14]. It was observed that “the double-membrane network of *G. obscuriglobus* cells emanates from the intracytoplasmic membrane to form unsealed compartments.” In that study, the bacteria were preserved close to native state, sectioned, and imaged under cryogenic conditions to reduce preparation-induced artifacts. However, because of the difficulties involved in sectioning cells under liquid nitrogen temperatures and the technical challenges presented by the use of vitrified sections in obtaining serial sections of a whole cell, the analysis was based on tomographic reconstruction of only a fraction, up to 150 nm thick sections, of *G. obscuriglobus* cells, which are usually ∼2 µm in diameter.

We have recently contributed to this series of analyses and have described the cell organization in two types of *G. obscuriglobus* cells [15]. In the first type, the dividing form, the inner membrane protrudes deeply into the cytoplasm to form thin membrane sheet invaginations extending towards the inside of the cell. The second cell type is not budding, and has increased periplasmic volume populated by vesicle-like structures.

Till present, how the membranes are organized in 3D is not known for any of the PVC bacteria. We have thus investigated the 3D membrane organization in multiple cells of the species *G. obscuriglobus*. In order to capture the membrane organization of entire cells, we chose to use plastic embedding for this study. Here we present the reconstructed volume of one complete cell of the first, dividing type, where we followed the entire organization of internal membranes within the cell. We report for the first time the 3D reconstruction of a bacterium with a complex endomembrane system. Our 3D reconstruction reveals that *G. obscuriglobus* cells are neither compartmentalized nor nucleated. We show that the spaces created by the membrane invaginations are all interconnected and not closed. The organization of cellular space is similar to that of a classical Gram-negative bacterium modified by the presence of large invaginations of the IM inside the cytoplasm.

## Results

We acquired tomograms from 10 different bacterial cells (Table S1). We encountered difficulties with attempts to automatically track membranes and their interconnections. Currently, there is no software available that can accurately assign and follow the membranes in such a complex system and our attempts at automation did not achieve satisfactory results. We therefore manually assigned and traced membranes in more than 200 slices. In addition, reconstruction and modeling in 3D also required some manual intervention.

### Extensive Membrane Organization

**Figure 1:**
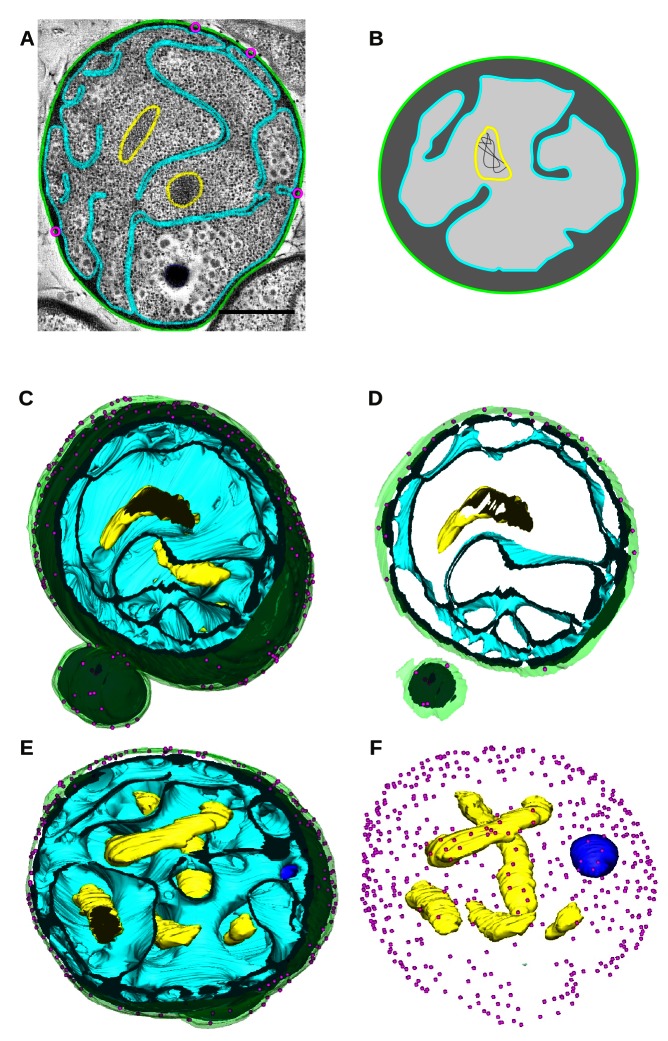
3D reconstruction of bacteria with a complex endomembrane system. (A) One slice of the tomogram is represented with the different structural features modeled. The OM is in green, the IM in cyan, the DNA in yellow, OM invaginations are in pink, and the Poly-P granule in dark blue. Scale bar is 500 nm. (B) Schematic of the cellular organization of *G. obscuriglobus*, not to scale. The periplasmic and cytoplasmic spaces are in dark and pale grey, respectively. Other colors are the same as in (A). (C and E) The modeled volume of one cell, sliced roughly through the middle, is represented in different orientations. Two views through the full volume are represented. (D) A slice through the same orientation as in panel (C) might give the false impression of a compartment surrounding the DNA. (F) Full volume representation without the membranes showing the five fragments of DNA, the crateriform structures, and the granule.

Three-dimensional reconstruction reveals that *G. obscuriglobus* cells have a cell plan that is not radically different from that of a typical Gram-negative bacterium (Figure 1; Figure S1). The organization is topologically compatible with an extension of the periplasmic space by invagination of the bacterial IM towards the cell's interior. This is supported by the fact that ribosomes line the IM and its invaginations in *G. obscuriglobus* cells, as they do along the IM of other Gram-negative bacteria. This similarity of topological organization is supported by genomic information [11]–[13]. The main difference is that the *G. obscuriglobus* IM invaginates extensively towards the interior of the cell to form a network of sheets within the cytoplasm (Figure 1; see Supplementary Movies 1 and 2, available at http://www.bork.embl.de/~devos/project/apache/htdocs/plancto/g3d/ [Text S1]). The space inside the invaginations is continuous with the periplasm and devoid of ribosomes, as in other bacteria (Figure 1; Figure S1).

We have observed ribosome-covered extended membrane sheets, as in the eukaryotic rough endoplasmic reticulum (ER) or the nuclear envelope, which have associated ribosomes, as opposed to membrane tubules associated to the eukaryotic smooth ER. The mean lumenal width of the internal membrane sheets is ∼20 nm (mean of 18.8). This is slightly smaller than the ∼30 nm and ∼50 nm reported, respectively, for yeast and mammalian ER sheets [16].

These membrane extensions have a significant impact on the cell organization, in particular on the ratio of OM versus IM. *E. coli* cells are about 1.5 µm long and 0.5–0.6 µm in diameter, with a cell volume of ∼0.65 µm^3^ [17]. Their periplasm comprises between 20% and 40% of the total cell volume [18]. With a diameter of ∼2 µm, the complete volume of the reconstructed *G. obscuriglobus* cell is 3.4 µm^3^, while the cytoplasm is 2.6 µm^3^. The periplasm, including the space created by the invaginations of the IM, has a volume of .82 µm^3^ (∼one third, 31.7%, of the cell's volume, similar to *E. coli*). The important difference is observed at the membrane surface. In *E. coli*, the IM/OM ratio is slightly below 1. In this particular *G. obscuriglobus* cell, the OM has a surface of 13.7×10^6^ nm^2^, while the IM is almost exactly three times bigger, with a surface of 42.7×10^6^ nm^2^ (Table S2). Based on our observations, this ratio likely varies from cell to cell.

### Absence of Individualized Compartments

Although extensively developed, the membrane does not create individualized compartments within the cytoplasm. All membranes are connected and isolated compartments defined by membranes within the cell volume do not exist. The only cellular volumes are the cytoplasm and the periplasm (Figure 1; see Supplementary Movies 1 and 2, available at http://www.bork.embl.de/~devos/project/apache/htdocs/plancto/g3d/ [Text S1]). *G. obscuriglobus* membrane invaginations and derived membrane morphologies appear to be dynamic and possibly cell-cycle-dependent [9],[15]. We have acquired partial volumes for six cells and complete volumes for four cells with various morphologies and believe it is highly unlikely that the membrane completely encloses or forms isolated compartments during any stage of the cell cycle. We have always observed connected pseudo-compartments that we could follow in 3D. The changes in membrane organization and connection of the pseudo-compartments, as well as the variation of periplasm organization, can be followed in consecutive slices from the tomograms (Figures S2, S3, S4, S5, S6, S7, S8).

### DNA

We observed five isolated clusters of DNA in one completely reconstructed cell and similar results in other cells (Figure 1; Figures S2, S3, S4, S5, S6, S7, S8). Some regions appeared more condensed than others, possibly due to differences in the replication or transcriptional status of the genetic material, which is unknown since the cell is in a dividing state.

Importantly, the genetic material is not restricted to a closed compartment with communicating pores—that is, in a “nucleus-like” organization as previously concluded [8],[10]. Membrane invaginations are sometimes found close to the DNA, but never enclose it completely. It is, however, easy to see why this can lead to false interpretations when looking at 2D images of single sections (Figure 1). 3D reconstruction rules these out. We have obtained similar tomograms for nine additional *G. obscuriglobus* cells with distinct overall membrane organization, and reached the same conclusion in each case (Figures S2, S3, S4, S5, S6, S7, S8). This conclusion is consistent with the presence of ribosomes in the cytoplasm surrounding the nucleoid. The DNA appears to be floating freely within the cytoplasm and does not obviously interact with the membranes, as in other bacteria.

### The Bud and the Neck

**Figure 2:**
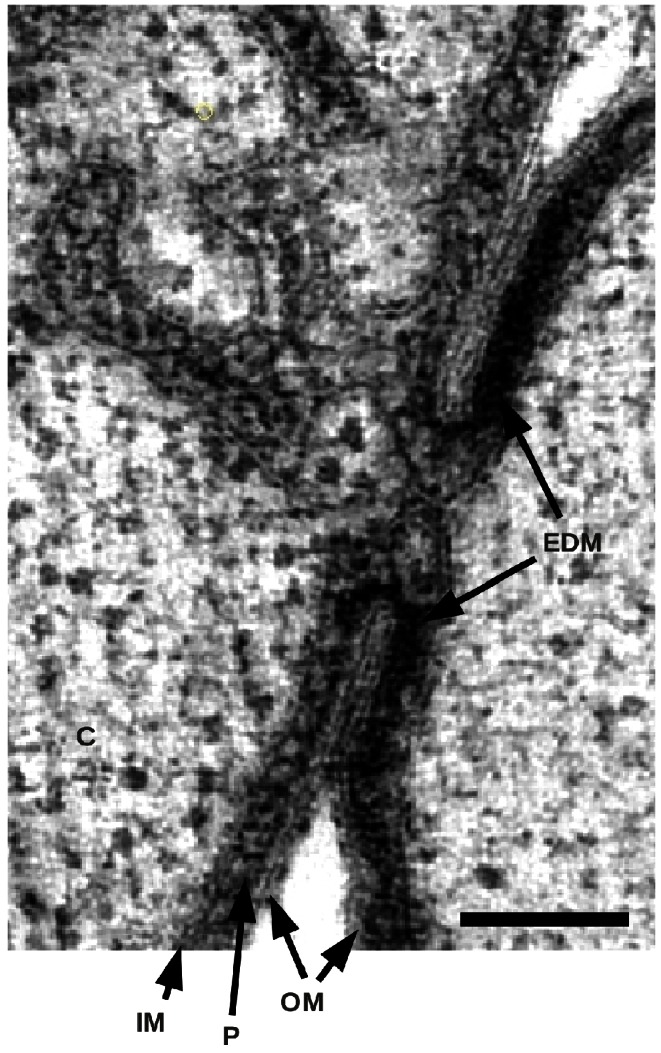
Membrane continuity between mother and daughter cells. Electron micrograph of the neck of the bud. Mother cell (left) and daughter cell (right). Electron dense material is present in the periplasm around the neck. Outer- (OM), inner-membrane (IM), cytoplasm (C) periplasm (P), and electron dense material (EDM) are indicated by arrows. Scale bar is 100 nm.

**Figure 3:**
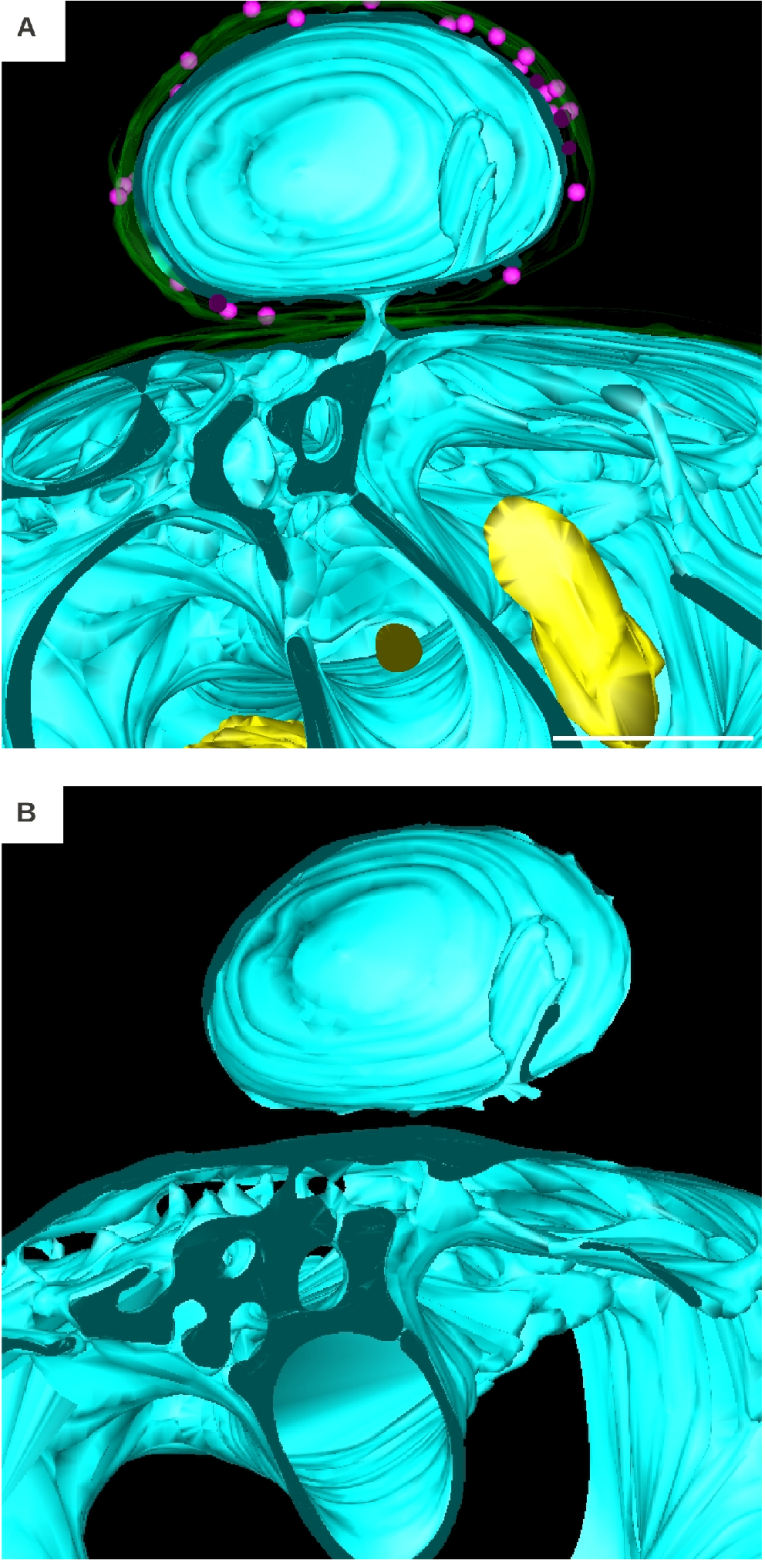
Membrane organization around the neck of the bud. (A) 3D model displaying membrane organization in proximity to the budding neck. All features are represented and color-coded as in Figure 1. The neck can be observed linking the mother cell (below) to the bud (above). (B) Only the IMs are represented in this image. The more complex organization of the IM can be observed in the mother cell; the single membrane sheet can be observed in the bud. Scale bar is 300 nm.

Almost all planctomycetes reproduce by budding [7], instead of fission, the most common form of bacterial division. During the early phases of the budding process, the bud is mostly devoid of membranes and DNA [9]. Consistently, we imaged the bud where only one membrane sheet is present and we do not detect any DNA. This membrane sheet is ∼20 nm thick, similar to those observed in the mother cell. Furthermore, the IM of the bud is continuous with the IM of the mother cell, as can be observed at the neck of the bud (Figure 2; see Supplementary Movie 3, available at http://www.bork.embl.de/~devos/project/apache/htdocs/plancto/g3d/ [Text S1]), implying continuity for all membranes between the mother and daughter cell. The cytoplasm of the mother and daughter cells are connected by a narrow channel through the neck of the bud. At its narrowest point, the channel is roughly 30 nm wide, explaining why it has been missed in previous studies. Moreover, electron dense material is observed inside the periplasm around the neck, possibly suggesting the periplasm as an alternative route for the transfer of material between the mother and the daughter cells. However, this dense material requires further study and confirmation. As the bud enlarges, the neck of the bud opens up, with dimensions ranging from 80 to 375 nm (Figure S9). The genetic material, being cytoplasmic, can pass freely into the bud without interference from a “nuclear membrane.” This structure must somehow close during completion of cell division. The membrane organization in the mother cell appears to become more complex in the proximity of the budding neck (Figure 3; see Supplementary Movie 3,, available at http://www.bork.embl.de/~devos/project/apache/htdocs/plancto/g3d/ [Text S1]), possibly due to the process of membrane transfer to the bud. However, also here, there are no defined compartments and all membranes are in continuity with the IM. These results have important implications for our understanding of planctomycete division.

### Crateriform Structures

**Figure 4:**
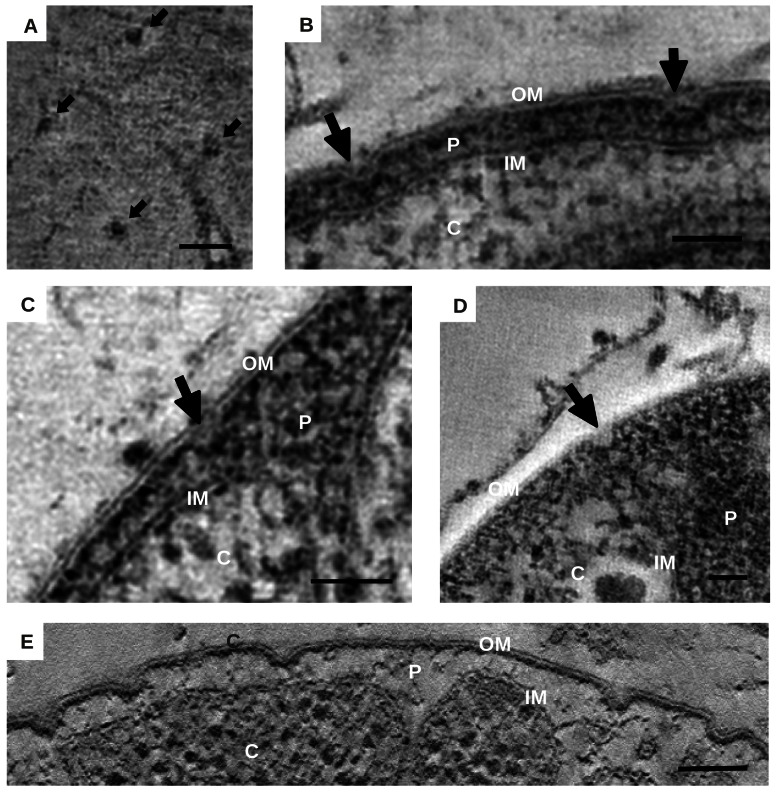
Crateriform structures. (A) Crateriform structures seen from the outside of the cell, indicated by arrows. (B–E) Micrographs of crateriform structures seen from the side, perpendicular to the membrane, indicated by arrows. Outer- (OM), inner-membrane (IM), cytoplasm (C), and periplasm (P) are indicated. Scale bars are 50 nm.

Crateriform structures have previously been reported as homogeneously distributed in *G. obscuriglobus* as opposed to other planctomycetes [19]. These structures are associated with depressions of the OM as can be seen from the side view perpendicular to the membrane (Figure 4). They have an opening of ∼35 nm and are uniformly distributed around the cell periphery, except in the mother cell within ∼1 µm diameter around the neck of the bud, with a density of between 50 and 100 crateriform structures per µm^2^ (Figure 1).

### Black Dot Granule

We have observed the presence of intracellular electron dense granules that are not enclosed by a membrane (Figure 1, depicted in dark blue). These are visible in roughly 50% of the cells that have been observed, and there is generally one per cell. X-ray micro-analysis confirmed that those granules are mainly composed of poly-phosphate (PolyP; Figure S10). PolyP can perform different biological functions, such as serving as an energy source for ATP synthesis [20].

## Discussion

Previously, there have been two related claims of the uniqueness for the planctomycetes and verrucomicrobiae compared to other bacteria [8],[21]. The first claim was the distinctive status of its membranes with the lack of an OM and thus of periplasm, the presence of an outermost cytoplasmic membrane, and an ICM. The second claim was linked to the organization of this ICM, stating that it divides the cytoplasm into compartments. Most importantly, the presence of a membrane surrounding the DNA in a structure related to the eukaryotic nucleus was postulated [7],[8],[21]. The uniqueness of the PVC membrane organization has recently been challenged by genomic information. This includes the presence of remnants of the *dcw* cluster, typical of Gram-negative bacteria, including peptidoglycan synthesis and cell division genes—for example, the otherwise ubiquitous *FtsZ* [13]. In addition, proteins typical for the OM and periplasm of Gram-negative bacteria are present in the genomes of PVC species [11],[12]. Using electron microscopy we can show, with confidence, that the second claim is not justified—that is, that the *G. obscuriglobus* cell plan is not compartmentalized and does not contain a nucleus-like structure. Importantly, our findings show that chromosomal DNA is not enclosed by a single membrane in *G. obscuriglobus*. This has important implications for our definition of eukaryotes and bacteria. Combined with the genomic evidence [11]–[13], this strongly supports the suggestion that the membrane organization of the PVC superphylum is not different from that of a Gram-negative bacterium, but an extension of it based on numerous invaginations of the IM. PVC species, with the possible exception of the anammox, do not have unique compartments; rather, their periplasm is extended by IM invaginations containing the two classical cell volumes, the periplasm and the cytoplasm. In addition this suggests that there is no spatial separation of transcription and translation by a membrane, as supported by the presence of ribosomes in close proximity to the DNA (Figure S11) [8],[15].

Our conclusion that the internal membranes of *G. obscuriglobus* cells result from a “simple” expansion of the periplasm by IM invaginations are likely to be applicable to other planctomycetes and other members of the PVC superphylum such as the verrucomicrobiae [22]. The presence of this extensive membrane organization in most PVC members suggests that, despite important variations, the ancestor of the PVC superphylum already had the precursor of this feature—that is, some capacity to invaginate its membranes [23]. Thus, our results expand our understanding of the bacterial cell plan without challenging it.

Until now, it was believed that the genomic material of *G. obscuriglobus* is enclosed in a membrane. This created a problem in explaining genome segregation when the cells undergo division and a satisfying solution has proven difficult to find [9]. Here we show that the duplicated DNA is free to transfer to the daughter cell without membrane interference, the only restriction being the width of the neck of the bud. Division is directly linked to the fact that all planctomycetes have lost the otherwise ubiquitous cell division protein FtsZ, while it is still present in *Lentisphaera* and *Verrucomicrobia* [13]. How PVC cells lacking FtsZ divide is unknown. So far, the only clue available is the detection of a GTPase-related novel cell division ring gene in the anammox bacteria, which is unrelated to FtsZ [24]. However, the anammox bacteria are divergent planctomycetes, and homologues of this protein have not been found in other planctomycetes, making it unlikely to provide a global answer to this question.

A particularity of the planctomycete membranes is that, with the exception of the anammoxosome, they have no assigned function [25]. Here we calculated that IM invaginations triplicate the surface of membranes relatively to the cell volume. Similar membrane extensions in photosynthetic bacteria are linked to the synthesis of energy. However, photosynthesis is not known to take place in *G. obscuriglobus*. Similar membrane extensions in eukaryotic cells include the mitochondria (linked to energy) and the ER/Golgi (linked to protein synthesis and secretion). It is interesting to compare the *G. obscuriglobus* endomembrane system with the eukaryotic endomembrane system. The eukaryotic rough ER and the outer membrane of the nuclear envelope are formed by membrane sheets that are coated with ribosomes, while the smooth ER is formed largely by membrane tubules devoid of ribosomes. In *G. obscuriglobus* cells, we have not observed tubules, only membrane sheets. As in the eukaryotic rough ER, ribosomes coat the sheets of the *G. obscuriglobus* endomembrane (Figure S11) [8],[15]. The function of the rough ER includes protein translocation into and through the ER lumen, as well as modification of newly synthesized secretory and membrane proteins. Smooth ER might be involved in lipid metabolism or Ca2+ signaling [16] and is specialized in sterol synthesis, a function also described in *G. obscuriglobus* [26]. Sterol modifies lipid fluidity and is thus linked to membrane organization [27]. The capacity to synthesize sterol is a feature previously considered as mainly eukaryotic. As opposed to the other sterol-producing bacteria, sterol synthesis in *G. obscuriglobus* is unlikely to be the result of a lateral gene transfer event from eukaryotes [26],[28]. Instead, it has been suggested that *G. obscuriglobus* could retain the most ancient remnants of the sterol biosynthesis pathway [26]. It seems likely that sterol synthesis in *G. obscuriglobus* is directly linked to the diversity of its extensive membrane organization. It is interesting to consider that protein composition can influence membrane bending [29]. In this respect it would be particularly interesting to investigate the membrane-bound proteins in planctomycetes.

### Evolutionary Considerations and the Origin of the Eukaryotic Cell

Eukaryogenesis has long been a question of major interest to biologists. Although it is increasingly accepted that eukaryotes and archaea share a common ancestor, the nature of this ancestor (if it was already an archaea per se or an intermediate organism) is still debated [30]. The eukaryotic cell is differentiated from bacterial and archaeal cells by many features whose origins are for the most part still unknown. These features include the actin- and tubulin-based cytoskeleton, the mitochondria, the nuclear pore, the spliceosome, the proteasome, and the ubiquitin signaling system [31]. Features reminiscent of these are increasingly detected in prokaryotes, including the members of the PVC bacterial superphylum [23],[32]. Because PVCs display some features related to eukaryotes or archaea, including sterol production [26] and ether-linked lipids [6], it has been proposed that the PVC ancestor might have shared a sisterhood relationship with the ancestor of the eukaryotes and archaea [23],[32]. Other scenarios involving a relationship between PVC and eukaryotes have also been proposed [21],[33]. However, whether the PVC features are homologous or analogous to their eukaryotic or archaeal counterparts is still under discussion [34]. If there is no evolutionary relationship between PVC and eukaryotes, the complex endomembrane system of those bacteria highlights that endomembrane systems have evolved more than once. The complex endomembrane system of *G. obscuriglobus* is in direct contact with proteins displaying structural similarities to eukaryotic membrane coat proteins like clathrin or sec31 that sustain the eukaryotic endomembrane system [15],[35]. In addition, *G. obscuriglobus* endomembranes are involved in the otherwise strictly eukaryotic process of endocytosis [36]. These data reinforce the possibility of an evolutionary relationship between the eukaryotic and PVC endomembrane systems and suggest that the latter could represent intermediary steps in the development of the former from a “classical” Gram-negative bacterium [23],. Deeper characterization of the PVC endomembrane system is therefore of great interest.

In conclusion, our analysis reveals that the membrane organization in *G. obscuriglobus* is not fundamentally different from that of “classical” bacteria, but a complex variant of it. The next step is to link those observations with the development of this endomembrane system in cells using live imaging methods.

## Materials and Methods

*G. obscuriglobus* cells were grown as previously described [15]. The cells were frozen in an HPM010 (Abra Fluid, Switzerland) high-pressure freezing machine and freeze substituted with either 1% Osmium tetroxide, 0.1% uranyl acetate, and 5% H2O and embedded in Epon or with 0.5% uranyl acetate and embedded in Lowicryl HM20. Thin (60 nm) and thick sections (250 nm) were placed on formvar-coated grids and post-stained with uranyl acetate and lead citrate. Thin sections were imaged on a CM120 Phillips electron microscope. For tomography, acquisition was done on a Technai F30 300 kv (FEI Company) microscope with dual axis tilt series (first axis from −60° to +60° with 1° tilt increment, second axis from −60° to +60° with 1.5° increment). We acquired nine serial sections and reconstructed them using fiducial gold particles with the weighted back projection algorithm. We joined consecutive serial sections using the etomo graphical user interface from IMOD (Boulder Laboratory for 3-D Electron Microscopy of Cells). We fully acquired four cells (eight to nine sections), two cells that are ∼75% complete (six sections), two cells that are ∼50% complete (four sections), and two cells that are ∼40% complete (three sections) (Table S1). The budding cell was modeled with IMOD and we traced the contours on at least every fifth slice over a range of 1,130 slices.

Tomograms have been deposited in the EMDB (http://www.ebi.ac.uk/pdbe/emdb/) under the accession numbers EMDB-2362 and EMDB-2363.

## Supporting Information

Figure S1 Comparison of bacteria with and without a complex endomembrane system. EM of *E. coli* (A) and *G. obscuriglobus* (B). One slice of the *G. obscuriglobus* tomogram is represented without (B) and with (E) the different cellular features modeled. In (E), the OM is in green, the IM in cyan, the DNA is surrounded in yellow, OM invaginations are pink spheres, and the Poly-P granule is surrounded in dark blue. Scale bar is 500 nm. Schematic of the cellular organization of *E. coli* (C) and *G. obscuriglobus*, (D) not to scale. The OM, IM, and the space between the two membranes (periplasm) are in dark grey, black, and pale grey, respectively.
(PDF)

Figure S2 Consecutive slices from tomograms of *G. obscuriglobus* cell 2. The organization of the membranes can be followed through the partial volume of the cell. Scale bar is 500 nm.
(PDF)

Figure S3 Consecutive slices from tomograms of *G. obscuriglobus* cell 3. The organization of the membranes can be followed through the partial volume of the cell. Scale bar is 500 nm.
(PDF)

Figure S4 Consecutive slices from tomograms of *G. obscuriglobus* cell 4. The organization of the membranes can be followed through the partial volume of the cell. Scale bar is 500 nm.
(PDF)

Figure S5 Consecutive slices from tomograms of *G. obscuriglobus* cell 5. The organization of the membranes can be followed through the partial volume of the cell. Scale bar is 500 nm.
(PDF)

Figure S6 Consecutive slices from tomograms of *G. obscuriglobus* cell 6. The organization of the membranes can be followed through the partial volume of the cell. Scale bar is 500 nm.
(PDF)

Figure S7 Consecutive slices from tomograms of *G. obscuriglobus* cell 7. The organization of the membranes can be followed through the partial volume of the cell. Scale bar is 500 nm.
(PDF)

Figure S8 Consecutive slices from tomograms of *G. obscuriglobus* cell 8. The organization of the membranes can be followed through the partial volume of the cell. Scale bar is 500 nm.
(PDF)

Figure S9 Variability of bud necks. Electron micrographs of various budding cells during the process of division. Scale bar is 1 µm.
(PDF)

Figure S10 Energy-dispersive X-ray microanalysis plots. (Top) X-ray microanalysis of an area in a *G. obscuriglobus* cell without an electron dense granule. (Bottom) Same analysis of an electron dense granule. The U peak is due to the uranyl-acetate stain.
(PDF)

Figure S11 Ribosomes along the membranes and around the DNA of *G. obscuriglobus*. Slice through an electron tomogram where areas are magnified apparently showing ribosomes on the membrane (surrounded by a red circle) or ribosomes freely floating in the cytoplasm (circled blue). DNA is surrounded by a yellow line. However, note that our micrographs are taken from thick sections where ribosomes are not as well visible as in thin sections. To illustrate this point better, we refer the readers to previous publications [8],[15].
(PDF)

Table S1 Number of electronic slices, acquired sections, and cell coverage in this analysis.
(DOC)

Table S2 Measurements of cell volumes and surfaces. ^a^(µm^3^). ^b^(×10^6^ nm^2^).
(DOC)

Text S1 Supplementary movie descriptions. Movies are available at http://www.bork.embl.de/~devos/project/apache/htdocs/plancto/g3d/.
(DOCX)

## Abbreviations

EM: electron microscopy
ER: endoplasmic reticulum
G−: Gram-negative
ICM: intracytoplasmic membrane
IM: inner membrane
OM: outer membrane
PVC: Planctomycetes, Verrucomicrobiae, and Chlamydiae
