# Supplementary figures and images for: Three-Dimensional Reconstruction of Bacteria with a Complex Endomembrane System
Source: PLoS Biol. 2013 May 21;11(5):e1001565. doi: 10.1371/journal.pbio.1001565 (PMC3660258; doi:10.1371/journal.pbio.1001565)

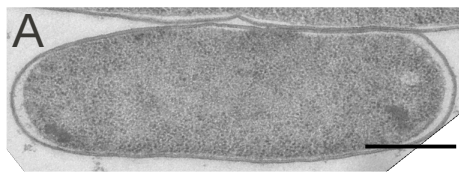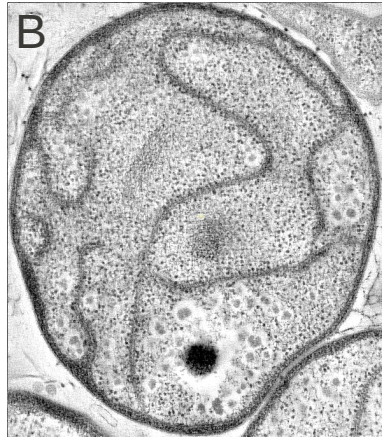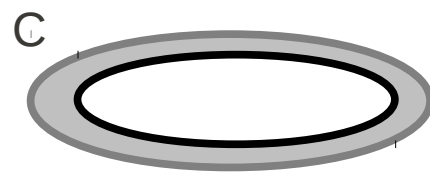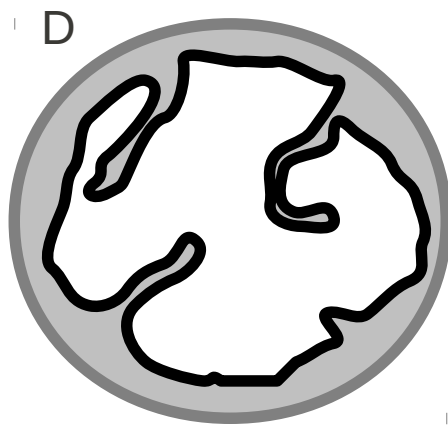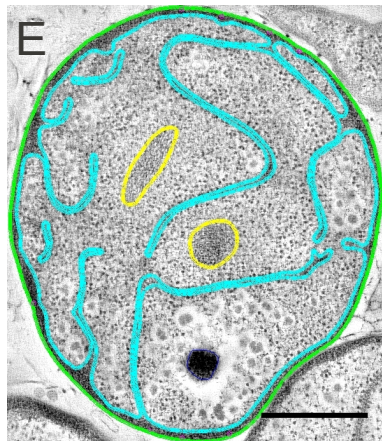

Supplement: Figure S1 — Comparison of bacteria with and without a complex endomembrane system. EM of E. coli (A) and G. obscuriglobus (B). One slice of the G. obscuriglobus tomogram is represented without (B) and with (E) the different cellular features modeled. In (E), the OM is in green, the IM in cyan, the DNA is surrounded in yellow, OM invaginations are pink spheres, and the Poly-P granule is surrounded in dark blue. Scale bar is 500 nm. Schematic of the cellular organization of E. coli (C) and G. obscuriglobus, (D) not to scale. The OM, IM, and the space between the two membranes (periplasm) are in dark grey, black, and pale grey, respectively. (PDF) [file pbio.1001565.s001.pdf]

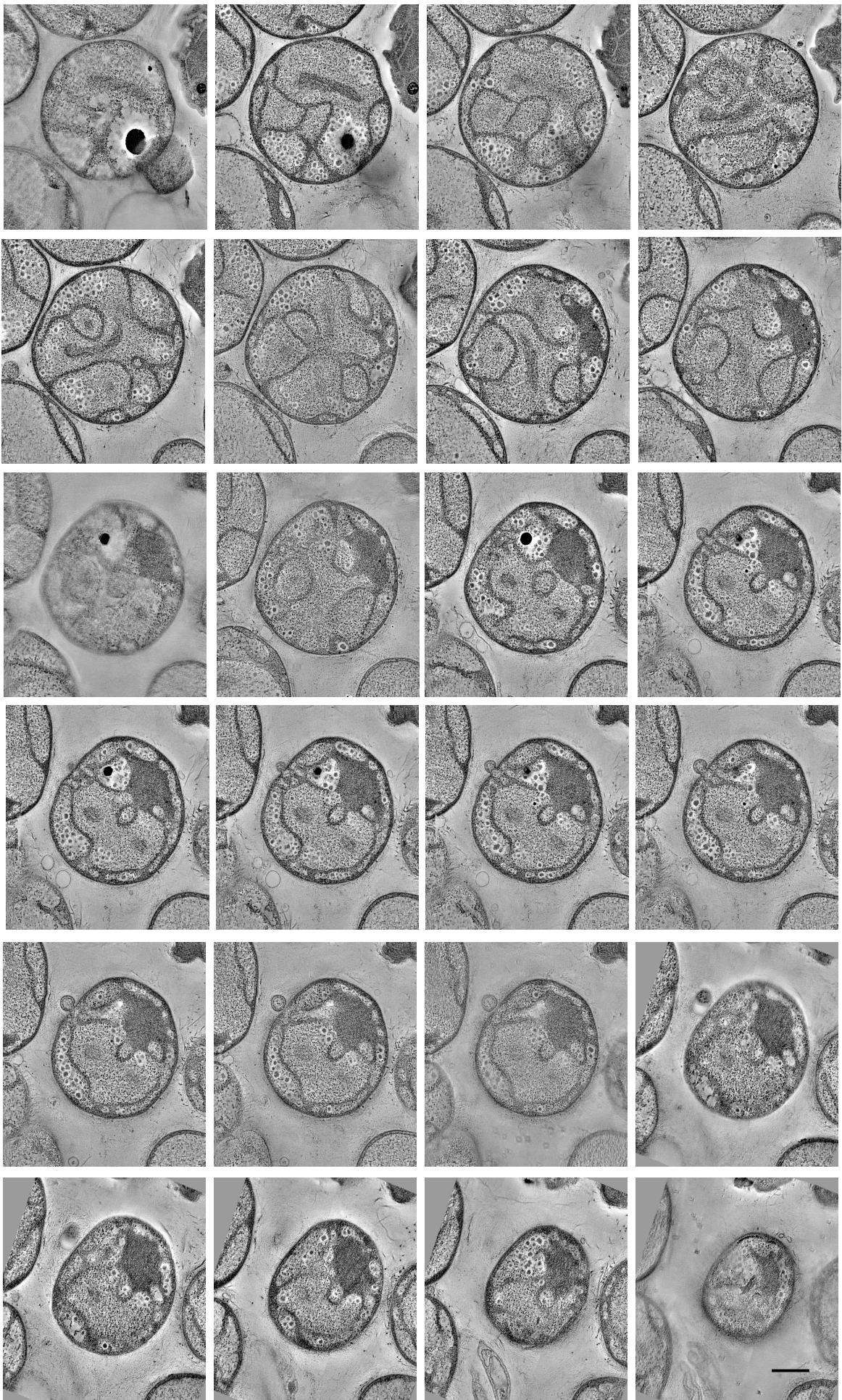

Supplement: Figure S2 — Consecutive slices from tomograms of G. obscuriglobus cell 2. The organization of the membranes can be followed through the partial volume of the cell. Scale bar is 500 nm. (PDF) [file pbio.1001565.s002.pdf]

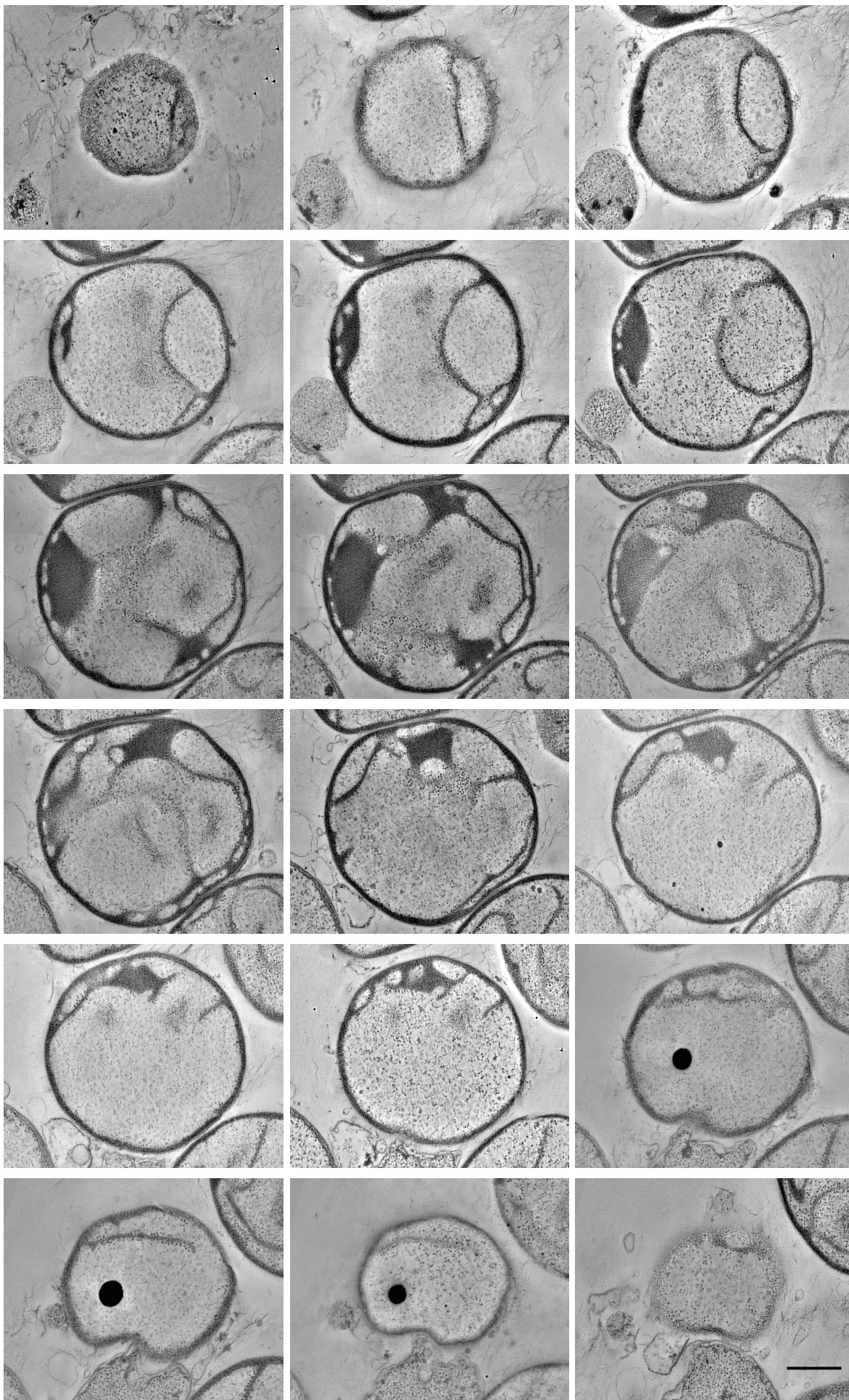

Supplement: Figure S3 — Consecutive slices from tomograms of G. obscuriglobus cell 3. The organization of the membranes can be followed through the partial volume of the cell. Scale bar is 500 nm. (PDF) [file pbio.1001565.s003.pdf]

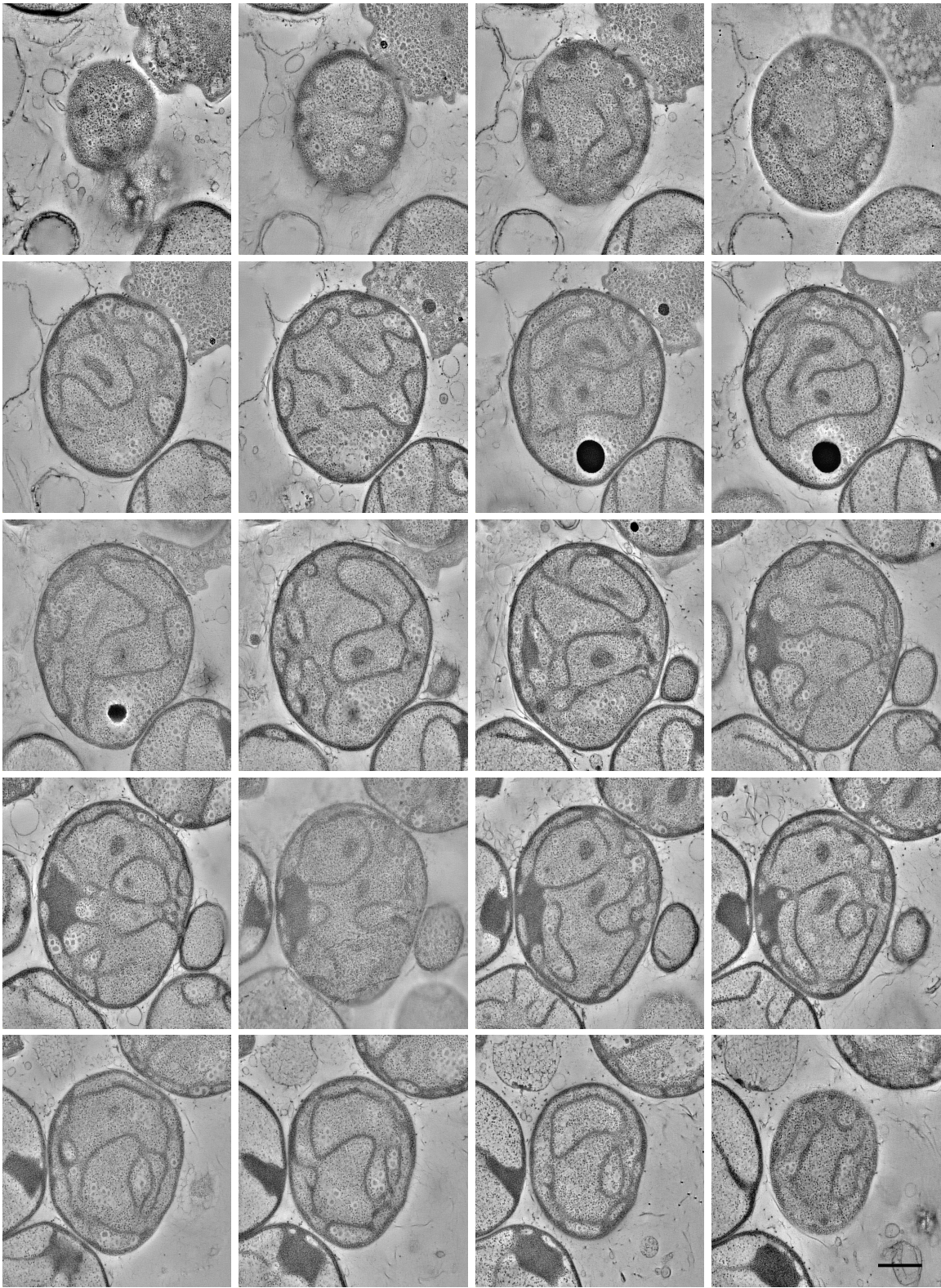

Supplement: Figure S4 — Consecutive slices from tomograms of G. obscuriglobus cell 4. The organization of the membranes can be followed through the partial volume of the cell. Scale bar is 500 nm. (PDF) [file pbio.1001565.s004.pdf]

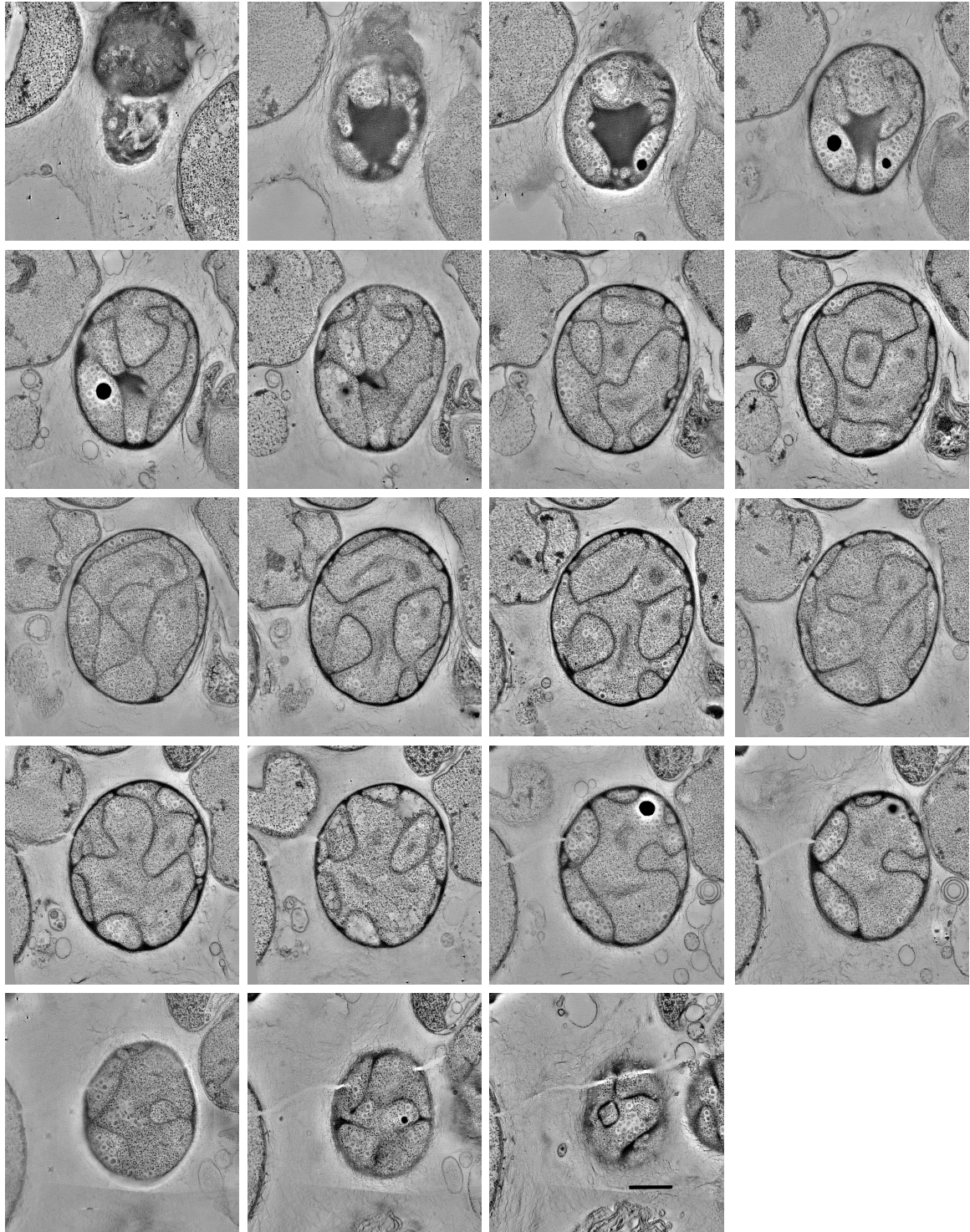

Supplement: Figure S5 — Consecutive slices from tomograms of G. obscuriglobus cell 5. The organization of the membranes can be followed through the partial volume of the cell. Scale bar is 500 nm. (PDF) [file pbio.1001565.s005.pdf]

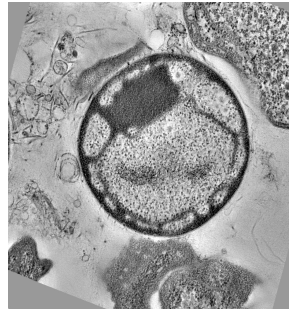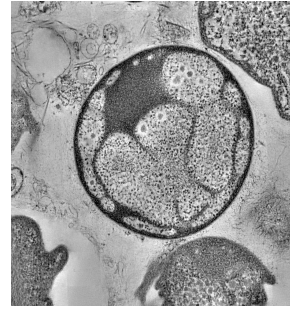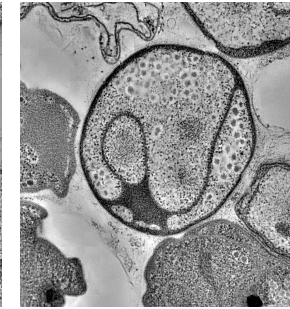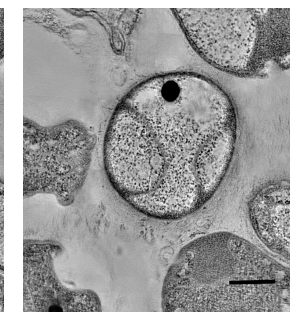

Supplement: Figure S6 — Consecutive slices from tomograms of G. obscuriglobus cell 6. The organization of the membranes can be followed through the partial volume of the cell. Scale bar is 500 nm. (PDF) [file pbio.1001565.s006.pdf]

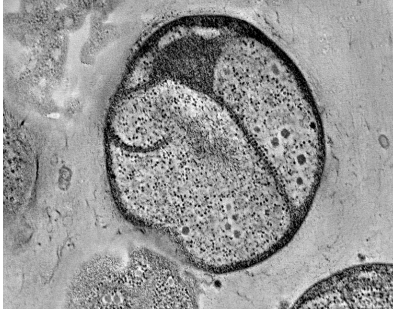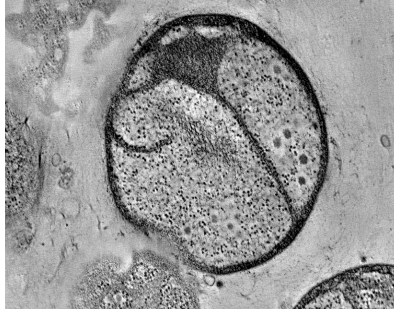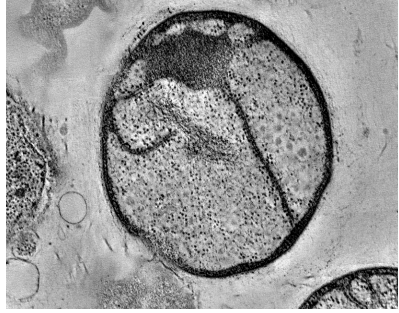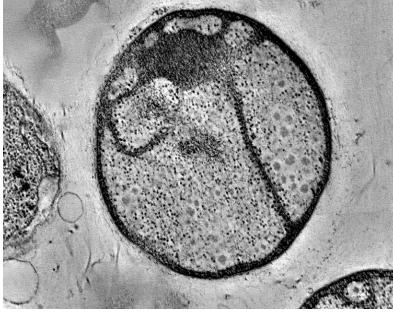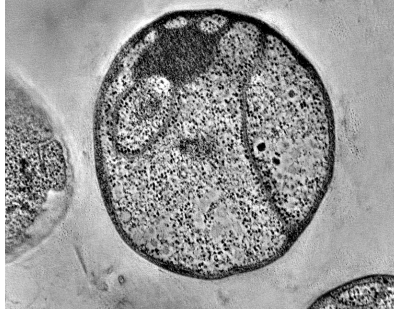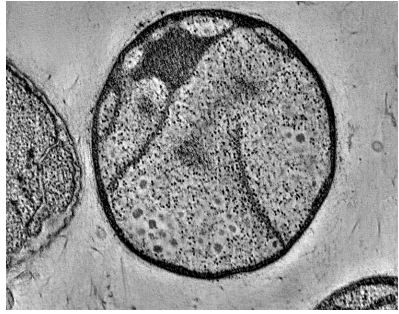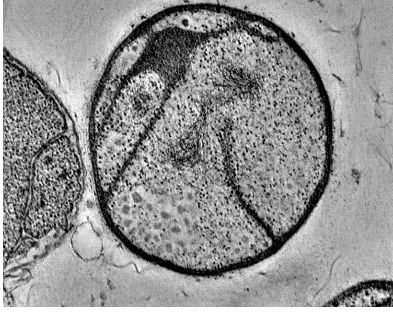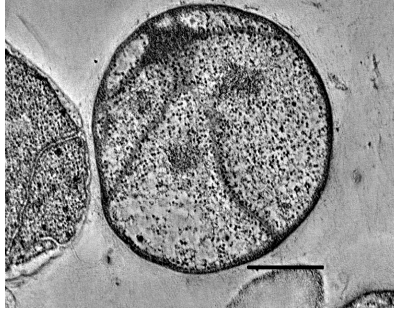

Supplement: Figure S7 — Consecutive slices from tomograms of G. obscuriglobus cell 7. The organization of the membranes can be followed through the partial volume of the cell. Scale bar is 500 nm. (PDF) [file pbio.1001565.s007.pdf]

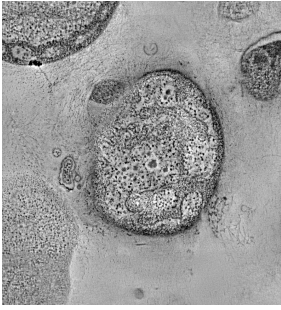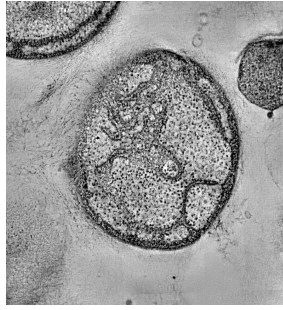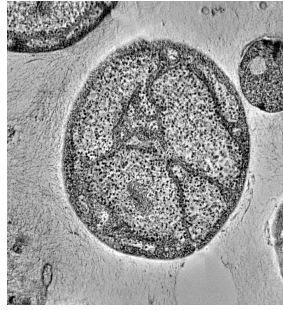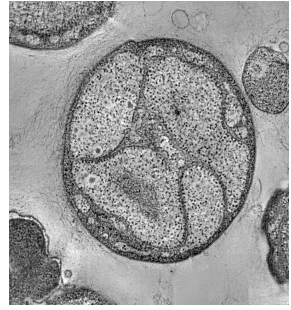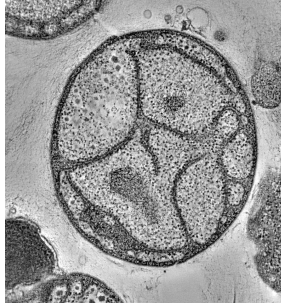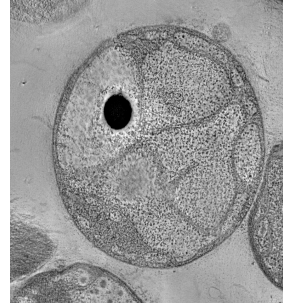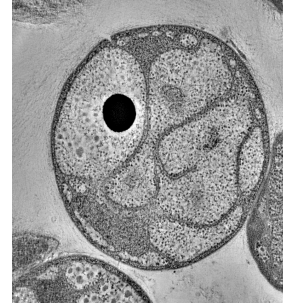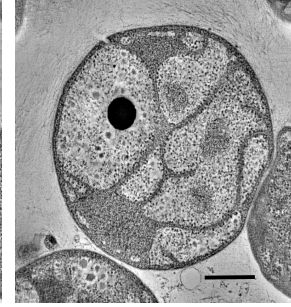

Supplement: Figure S8 — Consecutive slices from tomograms of G. obscuriglobus cell 8. The organization of the membranes can be followed through the partial volume of the cell. Scale bar is 500 nm. (PDF) [file pbio.1001565.s008.pdf]

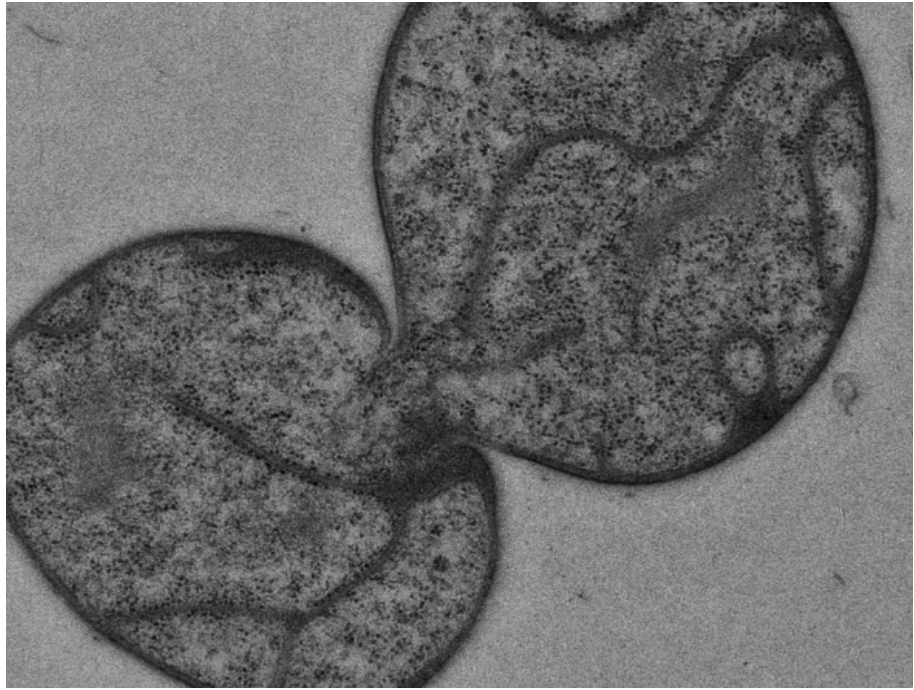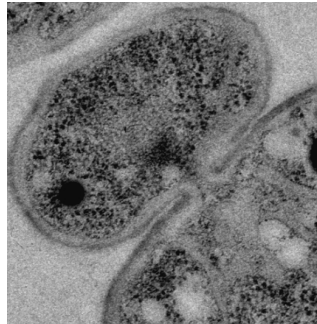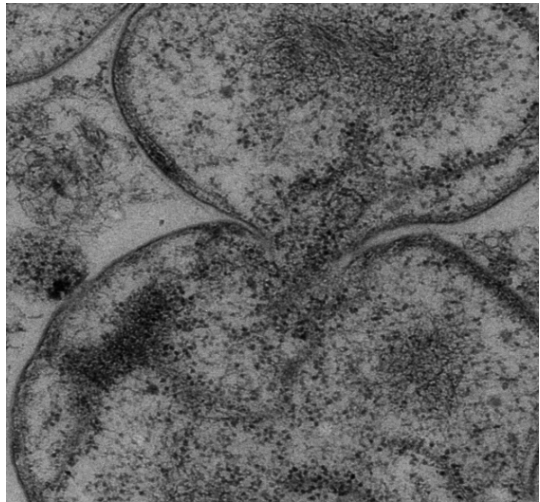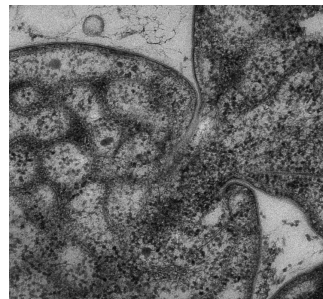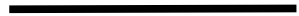

Supplement: Figure S9 — Variability of bud necks. Electron micrographs of various budding cells during the process of division. Scale bar is 1 µm. (PDF) [file pbio.1001565.s009.pdf]

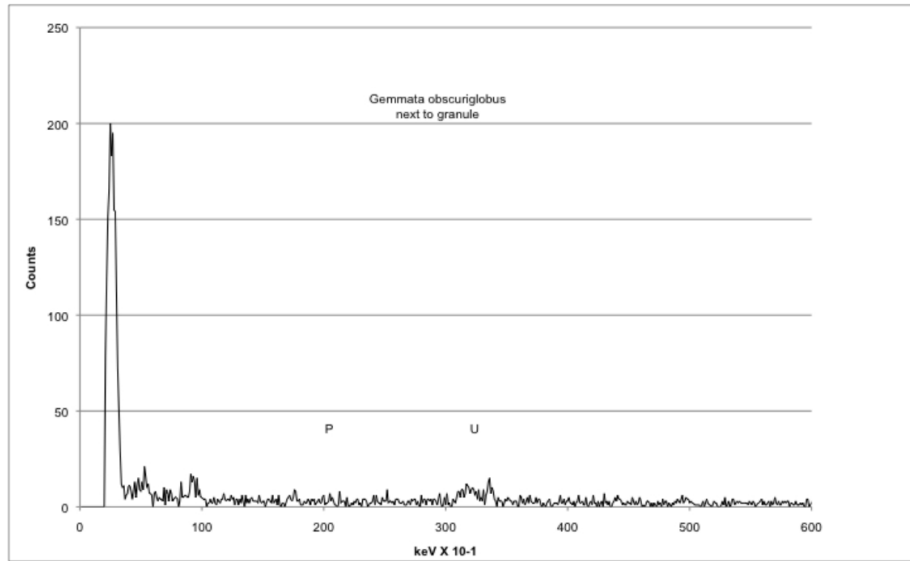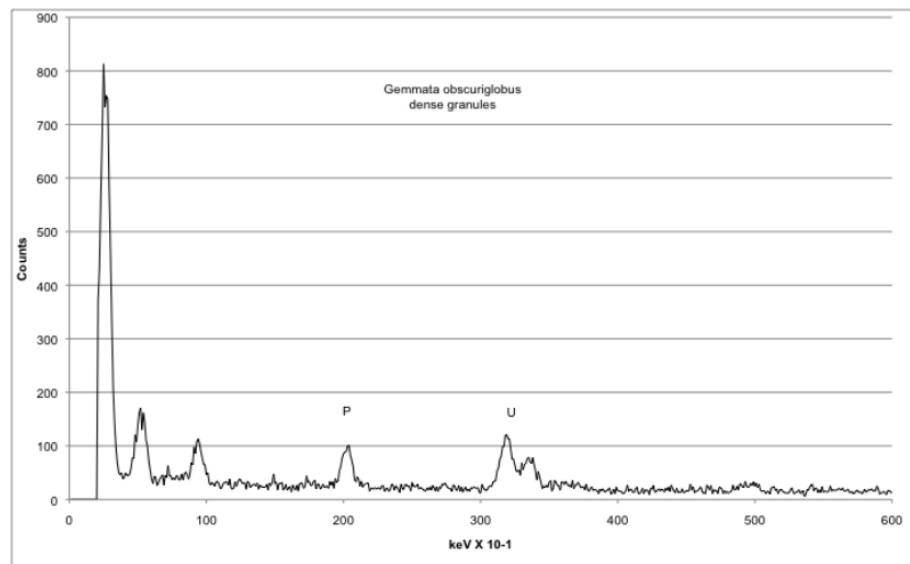

Supplement: Figure S10 — Energy-dispersive X-ray microanalysis plots. (Top) X-ray microanalysis of an area in a G. obscuriglobus cell without an electron dense granule. (Bottom) Same analysis of an electron dense granule. The U peak is due to the uranyl-acetate stain. (PDF) [file pbio.1001565.s010.pdf]

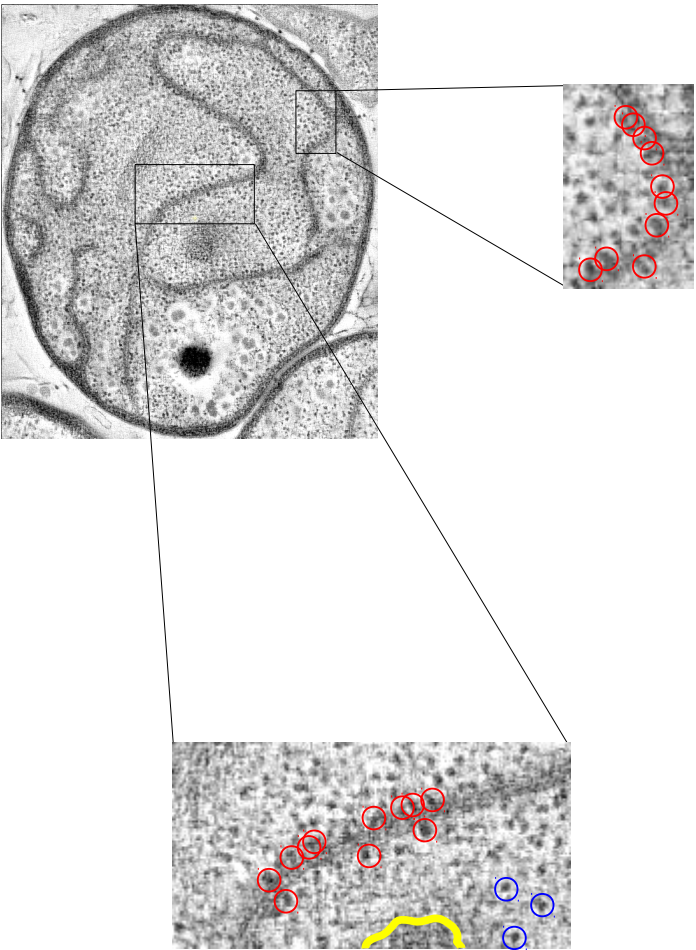

Supplement: Figure S11 — Ribosomes along the membranes and around the DNA of G. obscuriglobus. Slice through an electron tomogram where areas are magnified apparently showing ribosomes on the membrane (surrounded by a red circle) or ribosomes freely floating in the cytoplasm (circled blue). DNA is surrounded by a yellow line. However, note that our micrographs are taken from thick sections where ribosomes are not as well visible as in thin sections. To illustrate this point better, we refer the readers to previous publications [8],[15]. (PDF) [file pbio.1001565.s011.pdf]
