# Supplementary material for: Three-Dimensional Reconstruction of Bacteria with a Complex Endomembrane System
Source: PLoS Biol. 2013 May 21;11(5):e1001565. doi: 10.1371/journal.pbio.1001565 (PMC3660258; doi:10.1371/journal.pbio.1001565)
Supplement: Table S1 — Number of electronic slices, acquired sections, and cell coverage in this analysis. (DOC) [file pbio.1001565.s012.doc]

| Cell Identifier | Number of electronic slices | Number of acquired sections | Cell coverage |
| --- | --- | --- | --- |
| Cell 1 | 1069 | 8 | Full |
| Cell 2 | 1070 | 8 | Full |
| Cell 3 | 1161 | 9 | Full |
| Cell 4 | 964 | 8 | Full |
| Cell 5 | 879 | 6 | Almost full |
| Cell 6 | 850 | 6 | Almost full |
| Cell 7 | 547 | 4 | Half to 2/3^rd^ |
| Cell 8 | 573 | 4 | Half |
| Cell 9 | 409 | 3 | 1/3^rd^ |
| Cell 10 | 559 | 3 | 1/3^rd^ |
