# Supplementary material for: Three-Dimensional Reconstruction of Bacteria with a Complex Endomembrane System
Source: PLoS Biol. 2013 May 21;11(5):e1001565. doi: 10.1371/journal.pbio.1001565 (PMC3660258; doi:10.1371/journal.pbio.1001565)
Supplement: Table S2 — Measurements of cell volumes and surfaces. a(µm3). b(×106 nm2). (DOC) [file pbio.1001565.s013.doc]

|  | Total cell volume^a^ | Periplasm volume^a^ | Cytoplasm volume^a^ | OM*^b^* | IM*^b^* |
| --- | --- | --- | --- | --- | --- |
| *G. obscuriglobus* | 3.4 | 0.82 | 2.6 | 13.7 | 42.7 |
| *E. coli (Kubitschek, 1990)* | 0.6-.7 | 0.25 | 0.45 | 4 | 4 |
