# Supplementary material for: Three-Dimensional Reconstruction of Bacteria with a Complex Endomembrane System
Source: PLoS Biol. 2013 May 21;11(5):e1001565. doi: 10.1371/journal.pbio.1001565 (PMC3660258; doi:10.1371/journal.pbio.1001565)
Supplement: Text S1 — Supplementary movie descriptions. Movies are available at http://www.bork.embl.de/~devos/project/apache/htdocs/plancto/g3d/. (DOCX) [file pbio.1001565.s014.docx]

**Text S1. Supplementary movies.**

**Supplementary movies** are available at: <http://www.bork.embl.de/~devos/project/apache/htdocs/plancto/g3d/>.

**Supplementary Movie 1.** The movie scans through the tomogram and the membrane tracing of a full volume reconstruction of a *G. obscuriglobus* cell from two angles. Color code is as in Suppl. Fig. 1E. Scale bar is 500 nm.

**Supplementary Movie 2.** The movie scans through the tomogram and the membrane tracing of a full volume reconstruction of a *G. obscuriglobus* cell. The model is then visualize by cut-through under two different angles. Color code is as in Suppl. Fig. 1E. Scale bar is 500 nm.

**Supplementary Movie 3.** The movie scans through the model at the proximity of the neck of the bud. Mother cell is left, bud is right, outside is black. Color code is as in Suppl. Fig. 1E. The movie starts outside the bud, goes through the bud and comes out on the other side of the bud. The neck connecting the IM's is seen around the middle of the movie. Scale bar is 400 nm.
